# H_2_O_2_ sulfenylates CHE linking local infection to establishment of systemic acquired resistance

**DOI:** 10.1126/science.adj7249

**Published:** 2024-09-12

**Authors:** Lijun Cao, Sargis Karapetyan, Heejin Yoo, Tianyuan Chen, Musoki Mwimba, Xing Zhang, Xinnian Dong

**Affiliations:** 1 Department of Biology, Box 90338, Duke University, Durham, NC 27708, USA; 2 Howard Hughes Medical Institute, Duke University, Durham, NC 27708, USA

## Abstract

In plants, a local infection can lead to systemic acquired resistance (SAR) through increased production of salicylic acid (SA). For many years, the identity of the mobile signal and its direct transduction mechanism for systemic SA synthesis in initiating SAR have been debated. We found that, after a local infection, the conserved cysteine residue of the transcription factor CCA1 HIKING EXPEDITION (CHE) undergoes sulfenylation in systemic tissues, which enhances its binding to the promoter of SA-synthesis gene, *ISOCHORISMATE SYNTHASE1* (*ICS1*), and increases SA production. Furthermore, H_2_O_2_ produced through NADPH oxidases is the mobile signal that sulfenylates CHE in a concentration-dependent manner. Accumulation of SA and the previously reported signal molecules, such as N-hydroxypipecolic acid (NHP), then form a signal amplification loop to establish SAR.

## One Sentence Summary

H_2_O_2_ acts as a mobile signal for systemic SA synthesis and SAR by sulfenylating CHE.

Systemic acquired resistance (SAR) is an inducible immune mechanism in plants (*1–3*), triggered by a local immune response, providing long-lasting protection against a wide range of pathogens. This signaling phenomenon and its potential application in agriculture for engineering broad-spectrum disease resistance in crops have led to intense investigation and the discovery that de novo salicylic acid (SA) synthesis in systemic tissues is required for SAR (*4–6*). Though exogenous application of SA and its synthetic analogs has been shown to induce SAR without the presence of pathogens (*7–9*), for biological induction of SAR, how the local defense induces systemic synthesis of SA has remained a mystery. Multiple compounds, such as methyl salicylate, azelaic acid, dehydroabietinal, glycerol-3-phosphate (G3P), N-hydroxypipecolic acid (NHP), and extracellular nicotinamide adenine dinucleotide (phosphate) eNAD(P) (*10–16*), have been identified as SAR-inducing signals, with NHP most extensively studied and shown to function synergistically with SA (*3, 10, 15–23*), but none of them directly activates systemic SA synthesis.

## RESULTS

### CHE is required for systemic resistance

To identify the missing link between local pathogen infection and systemic SA production, we performed time-course measurements of several known SAR-inducing signals. Previous reports showed that the virulent *Pseudomonas syringae* pv. *maculicola* (*Psm*) ES4326 induced a significant NHP increase in uninoculated distal leaves 24 hours post inoculation (hpi), preceding SA induction at 48 hpi (*15, 20, 21, 24*), whereas when the avirulent *Psm* ES4326 strain expressing the effector, AvrRpt2, was used, a slight SA induction was detected at 24 hpi (25). To reconcile these results, we measured SA, NHP, and G3P in the same samples after mock or *Psm* ES4326/avrRpt2 treatment.

**Fig. 1.**
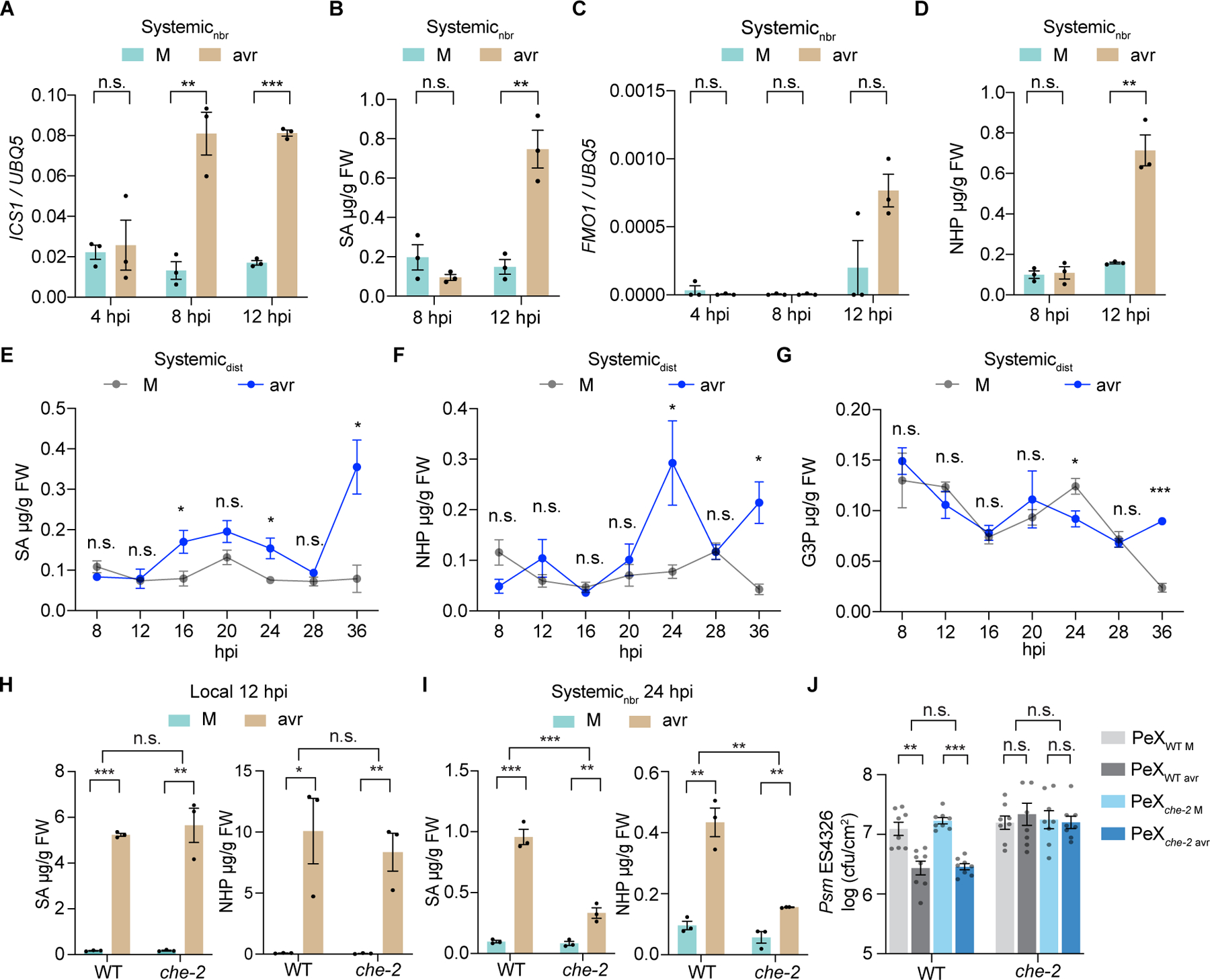
CHE is required for systemic SA and NHP production. (**A**-**D**) Time-course measurement of *ICS1* expression (A), SA level (B), *FMO1* expression (C), and NHP level (D) in the neighboring untreated half leaf (systemic_nbr_) tissues after infiltration of the other half with mock (M; 10 mM MgCl_2_) or *Psm* ES4326/avrRpt2 (avr; OD_600nm_ = 0.01). hpi, hours post infiltration. Data are means ± SEMs (*n* = 3). (**E**-**G**) Time-course measurement of SA (E), NHP (F), and G3P (G) in the un-inoculated distal leaf (systemic_dist_) tissues after local M or avr treatment. Data are means ± SEMs (*n* = 3). (**H** and **I**) Levels of SA and NHP in the treated half-leaves (local) (H) and systemic_nbr_ tissues (I) in wild type (WT) and the *che-2* mutant. Data are means ± SEMs (*n* = 3). (**J**) Bacterial growth in WT and *che-2* plants pre-treated with petiole exudate (PeX) collected from WT or *che-2* plants after M or avr treatment. Data are means ± SEMs (*n* = 8). Significant differences were calculated using either two-tailed Student’s t-tests or two-way ANOVA. ***P < 0.001; **P < 0.01; *P < 0.05; n.s., not significant.

Similar to A. Frank Ross’s pioneering SAR experiment in tobacco (1), we infected half-leaves of *Arabidopsis thaliana* with *Psm* ES4326/avrRpt2 and collected infected half-leaves as local and the uninfected half-leaves as neighboring systemic tissues (systemic_nbr_). We also infected two lower leaves with *Psm* ES4326/avrRpt2 and collected the upper uninfected leaves as distal systemic tissues (systemic_dist_). In systemic_nbr_ tissues, we observed an increased expression of the SA-synthesis gene *ISOCHORISMATE SYNTHASE1* (*ICS1*) at 8 hpi, preceding the induction of SA, the NHP-synthesis gene *FLAVIN-DEPENDENT MONOOXYGENASES* 1 (*FMO1*), and NHP at 12 hpi (Fig. 1, A to D). In systemic_dist_ tissues, a significant SA induction was detected at 16 hpi while increases in NHP and G3P were observed at 24 and 36 hpi, respectively (Fig. 1, E to G). Consistent with *ICS1* expression and SA production being regulated by the circadian clock (25), SA levels oscillated with a trough at 28 hpi. Detection of a robust SA induction earlier than NHP and G3P in distal tissues after the local *Psm* ES4326/avrRpt2 inoculation suggests that NHP and G3P are unlikely signals directly responsible for the initial systemic SA synthesis during SAR.

In searching for the direct regulator of systemic SA synthesis, we focused on the circadian clock transcription factor (TF), CCA1 HIKING EXPEDITION (CHE), which is required for both the basal circadian and pathogen-induced systemic SA production (25). To understand CHE’s regulatory role in SAR over time, we performed time-course RNA-mediated oligonucleotide Annealing, Selection, and Ligation with next-generation sequencing (RASL-seq) (26) to examine expression patterns of approximately 700 selected genes, primarily involved in defense and SA production. In local tissues, the *che* mutant showed similar overall gene expression patterns and levels of SA, NHP and its precursor pipecolic acid (Pip), and G3P compared to wild type (WT) (Fig. 1H, and fig. S1, A and B). This is consistent with that the *che* mutant maintains normal local defense (25). However, in systemic_nbr_ tissues, *che* displayed fewer transcriptional changes (fig. S1A) and substantially lower production of SA, NHP, and Pip (Fig. 1I, and fig. S1C) in response to SAR induction. However, the systemic G3P increase was unaffected in *che* (fig. S1C). These results show that CHE is required for SAR-associated gene expression and accumulation of SA and NHP only in systemic tissues. Interestingly, these CHE-mediated responses were not through transcriptional changes in *CALMODULIN BINDING PROTEIN 60g* (*CBP60g*) and *SAR DEFICIENT 1* (*SARD1*) involved in SA and NHP synthesis (*17, 27*), *CIRCADIAN CLOCK-ASSOCIATED 1* (*CCA1*), a clock gene targeted by CHE (28), or *RESPIRATORY BURST OXIDASE HOMOLOGUE D* (*RBOHD*), known for immune-induced apoplastic ROS production (*29–33*) (fig. S1D).

We next examined effects of *che* on the transportation and sensing of SAR-inducing signal(s) by measuring the activity of petiole exudates (PeX) (*34–36*). Notably, PeX from both WT and *che* after *Psm* ES4326/avrRpt2 treatment exhibited similar capacities in inhibiting bacterial growth in WT plants (Fig. 1J), suggesting that *che* can produce the SAR-inducing mobile signal(s) like WT. However, the inability of *che* to mount a defense in response to PeX-treatment indicates that it is defective in sensing the SAR-inducing signal(s). These findings further confirm that CHE is required for SA synthesis and resistance only in systemic tissues.

### Cysteine mutants of CHE are defective in SAR

**Fig. 2.**
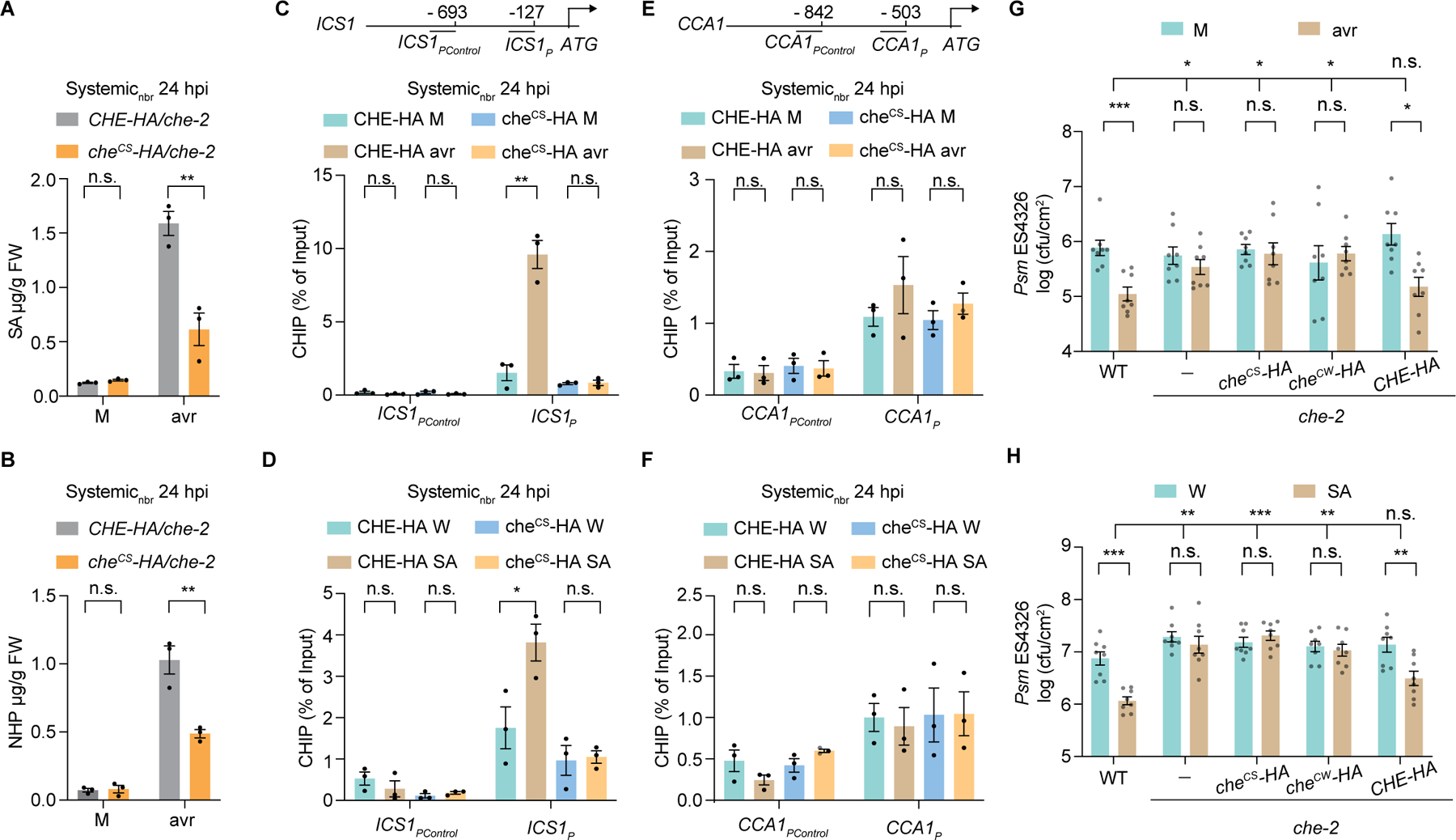
The cysteine residue in CHE is required for SAR. (**A** and **B**) SA (A) and NHP (B) in untreated half leaf (systemic_nbr_) tissues after mock (M; 10 mM MgCl_2_) or *Psm* ES4326/avrRpt2 (avr; OD_600nm_ = 0.01) treatments. *CHE-HA/che-2* and *che*^*CS*^*-HA/che-2*, WT CHE and the cysteine-to-serine mutant in *che-2* background, respectively. Data are means ± SEMs (*n* = 3). (**C** and **D**) ChIP-qPCR of CHE-HA and che^CS^-HA binding to *ICS1* promoter (*ICS1*_*P*_) after avr treatment (C), and after water (W) or SA treatment (D). Data are means ± SEMs (*n* = 3). (**E** and **F**) ChIP-qPCR of CHE-HA and che^CS^-HA binding to *CCA1* promoter (*CCA1*_*P*_). Data are means ± SEMs (*n* = 3). (**G**) Bacterial growth. Plants were infiltrated with M or avr two days before infecting distal leaves with *Psm* ES4326 (OD_600nm_ = 0.001), and bacterial growth was measured 3 days after the second infiltration. *che*^*CW*^*-HA/che-2*, the cysteine-to-tryptophan mutant. Data are means ± SEMs (*n* = 8). (**H**) Bacterial growth 1 day after SA treatment. Data are means ± SEMs (*n* = 8). Significant differences were calculated using either two-tailed Student’s t-tests or two-way ANOVA. ***P < 0.001; **P < 0.01; *P < 0.05; n.s., not significant.

Since neither *CHE* transcript nor protein level showed a significant increase in systemic_nbr_ tissues upon induction (fig. S2, A and B), we considered protein modifications as an activation mechanism. CHE, also named TCP21, is a Class I TCP (TEOSINTE BRANCHED1, CYCLOIDEA, and PCF) TFs with a conserved cysteine in the DNA-binding domain (37). Given that cysteine-containing TCP proteins are sensitive to redox conditions for their DNA-binding activity (38), and the single cysteine residue of CHE (cysteine 51) is conserved across plant species (fig. S2C), we mutated it and found that, unlike the WT CHE tagged with HA under its native promoter (*CHE-HA/che-2*), cysteine mutants, *che*^*CS*^*-HA/che-2* (cysteine to serine) and *che*^*CW*^*-HA/che-2* (cysteine to tryptophan), failed to complement *che-2* in inducing systemic *ICS1*, *FMO1,* and *PR1* (fig. S3A). Further analysis of the *che*^*CS*^*-HA* line revealed a similar defect in the expression of other SA synthesis-related genes *AVRPPHB SUSCEPTIBLE 3* (*PBS3*) and *ENHANCED DISEASE SUSCEPTIBILITY 5* (*EDS5*), except for the nonessential *ENHANCED PSEUDOMONAS SUSCEPTIBITY 1* (*EPS1*) (*39, 40*) (fig. S3B), and compromised induction of SA and NHP compared to the *CHE-HA* line (Fig. 2, A and B).

To examine the effect of the cysteine mutation on CHE’s transcriptional activity, we performed chromatin immunoprecipitation-quantitative PCR (ChIP-qPCR). In systemic_nbr_ tissues, we observed a significant increase in CHE-HA binding to the TCP-binding site (TBS) of *ICS1* promoter (*ICS1*_*p*_) compared to the control (*ICS1*_*PControl*_) carrying the SARD1-binding site, while this increase was compromised in *che*^*CS*^*-HA* transgenic lines (Fig. 2C). The *che*^*CS*^*-HA* mutant was also defective in response to exogenous SA treatment (Fig. 2D), consistent with the previous finding that *che* mutants are defective in SA-mediated SAR signal amplification (25). However, the binding of CHE to the TBS of *EDS5* promoter (*EDS5p*) was not affected by SAR induction (fig. S3C), nor was the promoter of *CCA1* (*CCA1p*) (Fig. 2, E and F) (28). Consistently, *che*^*CS*^*-HA* could rescue the short period phenotype of the *che-1 lhy-20* double mutant (28) (fig. S4, A and B). Additionally, we found that promoters of *AGD2-LIKE DEFENSE RESPONSE PROTEIN 1* (*ALD1*) and *FMO1* do not have known TBS or binding activity with CHE (fig. S5, A and B), suggesting that CHE does not directly regulate these genes. Further supporting that the cysteine residue of CHE modulates its binding to the *ICS1* promoter to activate systemic SA synthesis, neither *che*^*CS*^*-HA* nor *che*^*CW*^*-HA* could rescue the SAR deficiency of the *che* mutants (Fig. 2, G and H, and fig. S3D) (25).

### CHE is sulfenylated in an H_2_O_2_ concentration-dependent manner

**Fig. 3.**
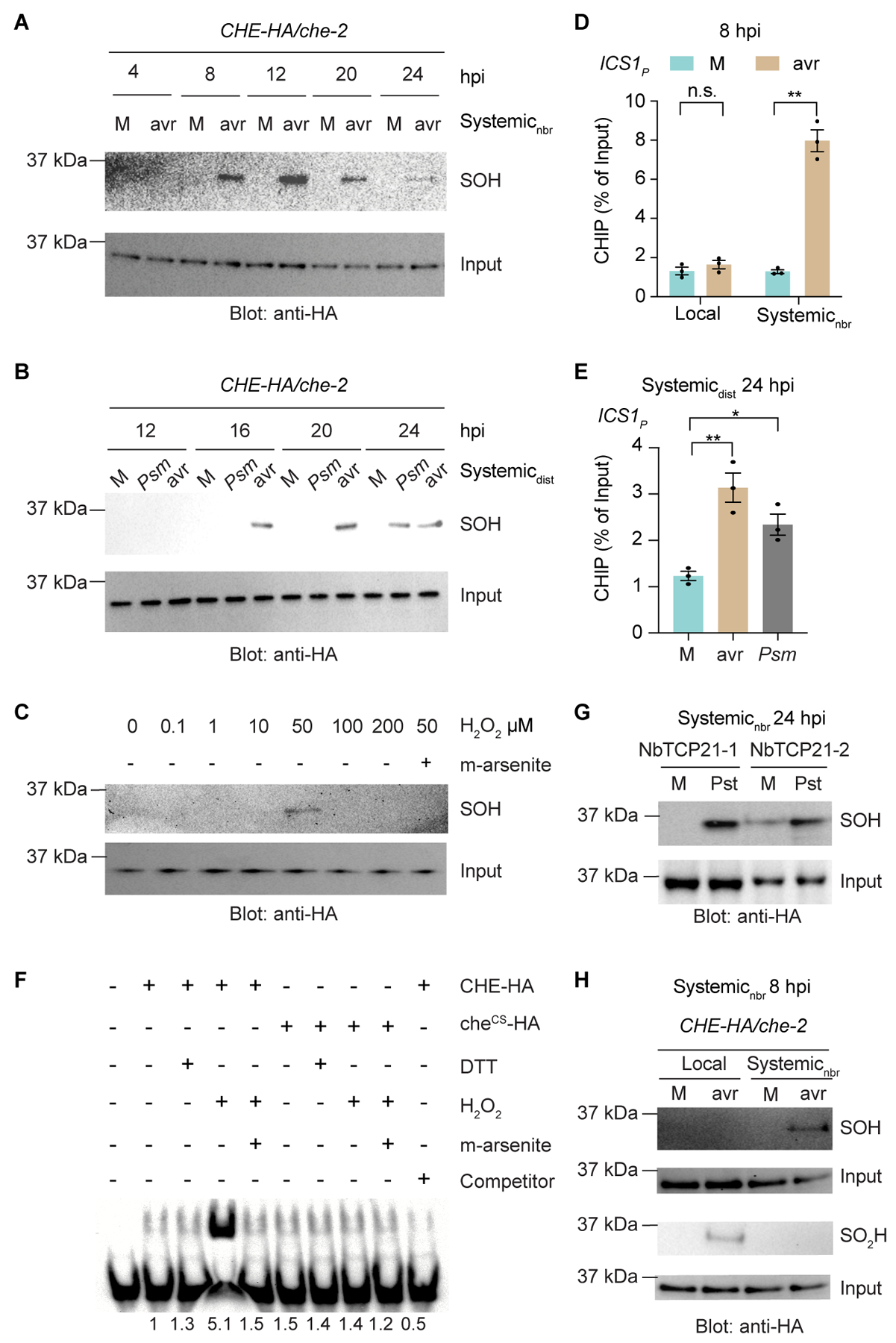
CHE-SOH occurs specifically in systemic tissues. (**A**) Sulfenylation (SOH) of CHE in the untreated half leaf (systemic_nbr_) tissues after mock (M; 10 mM MgCl_2_) or *Psm* ES4326/avrRpt2 (avr; OD_600nm_ = 0.01) treatment. *CHE-HA/che-2*, transgenic plants expressing WT CHE tagged with HA under its native promoter in *che-2* background. (**B**) CHE-SOH in the distal leaf (systemic_dist_) tissues triggered by avr or *Psm* ES4326 (*Psm*; OD_600nm_ = 0.01). (**C**) In vitro CHE-SOH after H_2_O_2_ treatment. Sodium arsenite (m-arsenite), a reducing agent that specifically removes sulfenylation. (**D** and **E**) ChIP-qPCR of CHE-HA binding to the *ICS1* promoter carrying the TCP-binding site (*ICS1*_*P*_) in the treated half-leaves (local), systemic_nbr_ (D) or systemic_dist_ (E) tissues. Data are the means ± SEMs (*n* = 3). (**F**) Electrophoresis mobility shift assay of recombinant CHE protein binding to the TBS-containing DNA probe from the *ICS1* promoter (*TBSi*). (**G**) Sulfenylation of *Nicotiana benthamiana* CHE homologs, NbTCP21-1 and NbTCP21-2 tagged by HA, in systemic_nbr_ tissues after M or *Pseudomonas syringae* pv. tomato DC3000 treatment of *N. benthamiana* plants. (**H**) SOH or Sulfinylation (SO_2_H) of CHE in local and systemic_nbr_ tissues. Significant differences were calculated using two-tailed Student’s t-tests. **P < 0.01; *P < 0.05; n.s., not significant.

To investigate how CHE initiates SAR, we focused on its cysteine residue. Since CHE has only one cysteine residue, a disulfide bond formation would require a cysteine-containing partner which was not detected using non-reducing SDS gel electrophoresis under mock or induced conditions (fig. S2B). We therefore performed biotin-switch assays (41) to capture CHE with other possible modifications by reactive oxygen species (ROS), such as S-sulfenylation (SOH), S-sulfinylation (SO_2_H) (42), and S-nitrosylation by reactive nitrogen species (*41, 42*) using modification-specific reducing agents. Notably, we found that CHE is sulfenylated (CHE-SOH), starting from 8 hpi in systemic_nbr_ tissues (Fig. 3A, and fig. S6A), consistent with the time of *ICS1* induction (Fig. 1A). In systemic_dist_ tissues, CHE-SOH was also detected, starting at 16 hpi following *Psm* ES4326/avrRpt2 inoculation (Fig. 3B), coinciding with SA increase in distal tissues (Fig. 1E). Inoculation with *Psm* ES4326 led to CHE-SOH at around 24 hpi (Fig. 3B). These findings show that CHE-SOH occurs earlier in neighboring tissues than in distal tissues, and faster after a local infection with *Psm* ES4326/avrRpt2 than with *Psm* ES4326. Additionally, exogenous SA application led to CHE-SOH at 24 hours post treatment (fig. S6B). To determine whether CHE can be sulfenylated in vitro, we treated purified CHE with various H_2_O_2_ concentrations, as H_2_O_2_ is produced during defense responses and induces microscopic cell death in systemic tissue to enhance resistance (*33, 43–45*). Interestingly, we found that CHE is sulfenylated specifically at around 40 to 50 μM H_2_O_2_, while lower or higher concentrations did not result in this modification based on the biotin-switch results (Fig. 3C, and fig. S6C).

To determine whether the enhanced CHE-binding to the *ICS1* promoter (Fig. 3, D and E) is due to sulfenylation, we performed a gel electrophoresis mobility shift assay using a DNA probe containing the *ICS1* TBS (*TBSi*). We found that treatment with 50 μM H_2_O_2_ significantly enhanced CHE binding to *TBSi* in vitro (Fig. 3F). This increase was due to CHE-SOH because treatment with m-arsenite, a reducing agent specific for SOH, diminished the binding. Moreover, the H_2_O_2_-induced binding was largely absent in che^CS^-HA mutant protein, confirming that sulfenylation of CHE is the molecular switch that increases its binding to the *ICS1* promoter. Additionally, we observed increased sulfenylation of *Nicotiana benthamiana* CHE homologs, NbTCP21-1 and NbTCP21-2 (46), after treatment with *Pseudomonas syringae* pv. tomato DC3000 (*Pst*) (Fig. 3G), an avirulent pathogen for *N. benthamiana (47)*. These results suggest CHE-sulfenylation is a conserved mechanism for systemic *ICS1* induction and SA synthesis during SAR.

To understand why CHE-SOH is only detected in systemic tissues, we hypothesized that higher ROS levels in local tissues might further oxidize the cysteine residue in CHE. We found that this was indeed the case because while sulfenylation (CHE-SOH) was observed in systemic tissues, sulfinylation (CHE-SO_2_H) was detected in local tissues (Fig. 3H) using the reacting agent specific for sulfinic acid, DiaAlk (48). Regardless of whether CHE-SO_2_H can be further oxidized to CHE-SO_3_H in local tissues, neither form is active in binding to the *ICS1* promoter (Fig. 3D). This H_2_O_2_-concentration-specific activation of CHE may also explain the controversy over H_2_O_2_’s role as a defense signal in previous studies due to differences in H_2_O_2_ concentrations used (*49, 50*).

### H_2_O_2_ serves as a mobile signal for SAR

**Fig. 4.**
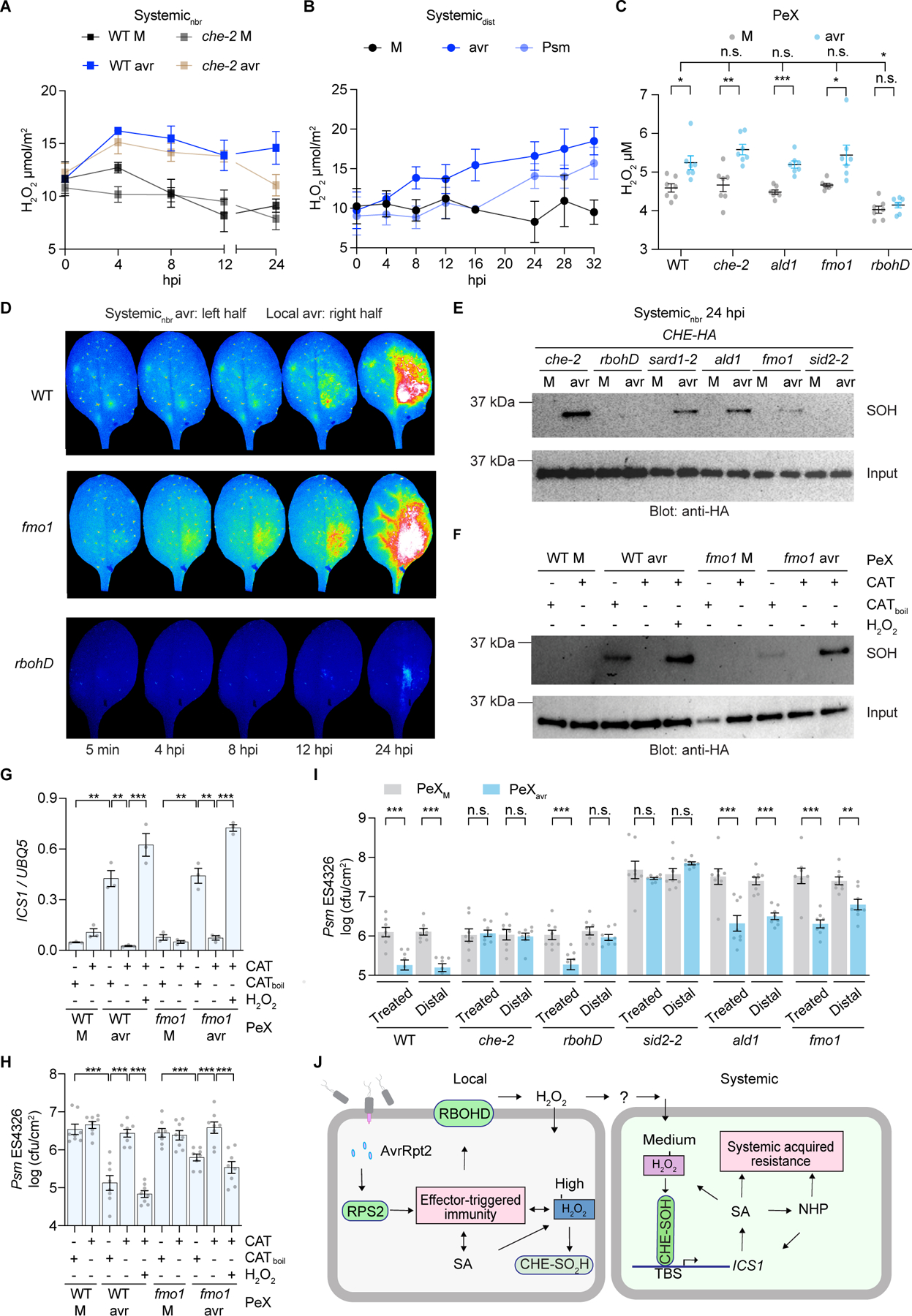
H_2_O_2_ initiates SAR. (**A and B**) H_2_O_2_ production in the untreated half leaf (systemic_nbr_) (A) or distal leaf (systemic_dist_) (B) tissues. Data are means ± SEMs (*n* = 5). (**C**) PeX H_2_O_2_ levels. Data are means ± SEMs (*n* = 7). (**D**) Snapshots of ROS imagings. (**E**) Sulfenylation (SOH) of CHE. (**F**-**H**) The PeX’s effect on CHE-SOH (F), *ICS1* expression (G), and bacterial growth (H) in WT plants. CAT_boil_, boiled catalase. Data are means ± SEMs, *n* = 3 for (G), *n* = 8 for (H). (**I**) Bacterial growth in the PeX-treated or distal leaves. Data are means ± SEMs (*n* = 8). (**J**) The model. Local defense results in high H_2_O_2_ accumulation and CHE sulfinylation (CHE-SO_2_H) without increasing its *ICS1* promoter-binding activity. Local defense activates RBOHD to produce H_2_O_2_ which accumulates to a moderate level in systemic tissues to sulfenylate CHE, enhancing CHE’s binding to the *ICS1* promoter to induce systemic SA synthesis and initiate SAR. Full-scale resistance is achieved through the synergistic activities of SA, NHP, and perhaps other signals. Significant differences were calculated using either two-tailed Student’s t-tests or two-way ANOVA. ***P < 0.001; **P < 0.01; *P < 0.05; n.s., not significant.

Since in vitro H_2_O_2_ treatment enhanced CHE’s binding to the *ICS1* promoter (Fig. 3, C and F), we investigated H_2_O_2_ as an endogenous signal for activating CHE by measuring H_2_O_2_ levels in systemic_nbr_ and systemic_dist_ tissues after induction by *Psm* ES4326/avrRpt2 and *Psm* ES4326. As expected, higher H_2_O_2_ induction was detected in the local tissues (~ 20 μmol/m^2^) compared to systemic_nbr_ tissues (~ 15 μmol/m^2^) from 4 hpi (Fig. 4A, and fig. S7A). Interestingly, this increase was not significantly affected in *che*, except at 24 hpi (Fig. 4A), nor in the *ald1* or *fmo1* mutants, but abolished in the *SALICYLIC ACID INDUCTION DEFICIENT 2* (*sid2*) mutant of *ICS1 (51)* and in *rbohD* (fig. S7, B and C). In systemic_dist_ tissues, H_2_O_2_ production started to increase later, at around 12 hpi for *Psm* ES4326/avrRpt2 and 24 hpi for *Psm* ES4326 (Fig. 4B). H_2_O_2_ levels in PeX collected from *che* and NHP-deficient *ald1* and *fmo1* mutants increased similarly to WT after infection, while no increase was detected in *rbohD* (Fig. 4C), demonstrating that H_2_O_2_ production in PeX requires RBOHD, but not NHP. Moreover, SA and NHP treatments could induce H_2_O_2_, similar to pathogen treatment (fig. S8, A and B). However, the NHP-mediated H_2_O_2_ production and subsequent CHE-SOH were abolished in the *sid2* mutant (fig. S8, B and C), indicating dependency on SA and consistent with previous reports of an amplification loop involving SA, NHP, and H_2_O_2_ (*20, 21, 49, 52*).

To monitor ROS (e.g., H_2_O_2_) transport from local to systemic tissues during SAR induction, we employed a live imaging technique using 2’,7’-dichlorodihydrofluorescein diacetate (*53, 54*). Upon *Psm* ES4326/avrRpt2 inoculation, ROS signals were detected in both WT and *fmo1* plants in local and, subsequently, in systemic_nbr_ tissues (Fig. 4D, and Movies S1 and S2), indicating this ROS signaling is independent of NHP production. To measure this signaling process at the whole plant level, we employed a luciferase reporter driven by the ROS-responsive promoter of *GLUTAREDOXIN 13* (*GRXS13*) (55). After *Psm* ES4326/avrRpt2 challenge, ROS accumulated in systemic_nbr_ and systemic_dist_ tissues at approximately 8 hpi and 16 hpi, respectively (Movies S3 and S4), aligning with the time of detection for H_2_O_2_ and CHE-SOH (Figs. 3, A and B and 4, A and B), but before the reported detection of other mobile signals, such as G3P, AzA, DA, and NHP (*12–15, 20, 21, 23, 56*). When *Psm* ES4326 was used as the inducer, ROS accumulation in the systemic_nbr_ and systemic_dist_ tissues occurred at approximately 12 hpi and 20 hpi, respectively, delayed by several hours compared with *Psm* ES4326/avrRpt2 challenge (Movies S5 and S6). Consistent with the measurements (Fig. 4C and fig. S7, B and C), ROS production and transport were compromised in the *rbohD* mutant (Fig. 4D, and Movie S7). RBOHD and its homolog RBOHF catalyze H_2_O_2_ production by transferring electrons to oxygen to form superoxide anion (O_2_^-^) which is converted to H_2_O_2_ (57). In support of an essential role of RBOHD and RBOHF in initiating SAR, the *rbohD* mutant is deficient in CHE-SOH, CHE binding to the *ICS1* promoter, *ICS1* induction, and, along with *rbohF*, in SAR (Fig. 4E and fig. S9, A to C). These findings demonstrate that NADPH oxidase RBOHD/F-produced H_2_O_2_ is an initiating mobile signal for establishing SAR.

### PeX H_2_O_2_ initiates SA synthesis in establishing SAR

To demonstrate that H_2_O_2_ is the signal that sulfenylates CHE and establishes SAR in systemic tissues, we measured SA, NHP, and G3P in PeXs collected from WT and *fmo1*. After *Psm* ES4326/avrRpt2 induction, NHP levels significantly increased in WT PeX but not in *fmo1* (fig. S9D). Additionally, a slight G3P elevation was also detected, whereas SA was undetectable in PeX from both WT and *fmo1*, confirming that SA is synthesized de novo in systemic tissues (5). To demonstrate H_2_O_2_ is the initial mobile signal for systemic SA synthesis, we treated PeX collected from both WT and *fmo1* with catalase (CAT) to deplete H_2_O_2_ (fig. S9E) and found that it abolished PeX’s ability to sulfenylate CHE (Fig. 4F), induce *ICS1*, *FMO1,* and *PR1* genes (Fig. 4G and fig. S9, F and G), and enhance resistance against *Psm* ES4326 in WT plants (Fig. 4H). This inhibitory effect of catalase was eliminated by heat denaturation (CAT_boil_) and overcome by adding back H_2_O_2_ after removing catalase. Importantly, PeX from *fmo1*, lacking NHP (fig. S9D), still elicited significant SAR responses, albeit slightly less than WT (Fig. 4H, and fig. S9F), consistent with previous reports that PeX from the NHP-deficient *ald1* mutant could still trigger significant systemic defense (*58, 59*). These results further support that H_2_O_2_ is required for CHE activation through sulfenylation to initiate systemic SA synthesis, while NHP contributes to SA accumulation through a mutual amplification loop (*20, 21*). Consistently, CHE-SOH was detected, with a reduction, in mutants of *ALD1*, *FMO1*, and *SARD1* (Fig. 4E) whose own expression is SA-inducible (*17, 27*), confirming their role in the amplification loop. Furthermore, NHP-induced SAR was found to be dependent on RBOHF-produced ROS and eNAD(P) (10), and treating plants with NAD(P) increased both H_2_O_2_ production and CHE-SOH (fig. S9, H and I).

Interestingly, while SAR-competent PeX could activate defense in both treated and distal leaves of WT plants, it failed to trigger the response in distal leaves of *rbohD* (Fig. 4I), indicating that RBOHD is required for the relay-production of H_2_O_2_ to induce SAR. Unlike *rbohD*, *che* and *sid2* were nonresponsive to PeX in either treated or distal tissues (Fig. 4I), supporting our conclusion that, in systemic tissues, CHE-mediated *ICS1* induction is required for SAR initiation and establishment. As expected, the *ald1* and *fmo1* mutants retained partial responsiveness to PeX, while displaying a defect in basal resistance, in contrast to the *che* and *rbohD* mutants (grey bars in Fig. 4I).

## Discussion

Our study revealed that NADPH oxidase-generated H_2_O_2_ is the primary signal initiating SAR by sulfenylating CHE and inducing de novo SA production in systemic tissues (Fig. 4J). This initial SA increase has likely been overlooked in earlier studies due to its relative subtelty compared to the large SA increase due to the amplification loop. By using timecourse measurements as well as live imaging (Figs. 1E and 4B, and Movies S1-S6), we were able to capture these early signaling events. It will be interesting to investigate whether and how NHP, G3P, eNAD(P) (10), and nitric oxide (*59–62*) are involved in the relay-production of H_2_O_2_ in coordination with RBOHD. Our study also lays the groundwork for exploring how systemic H_2_O_2_, SA, NHP, and other signals form an amplification loop to confer full-scale SAR (Fig. 4I). Though the complete circuitry of this amplification loop has yet to be elucidated (Fig. 4J), one study showed that SA can increase H_2_O_2_ production by inhibiting catalase activity, resulting in sulfenylation of tryptophan synthetase β SUBUNIT 1 to suppress auxin biosynthesis and confer resistance (52).

While “necrotizing pathogens” were initially used in the search for ways to “vaccinate” plants and trigger SAR (1), virulent pathogens, such as *Psm* ES4326, could also induce SAR (*10, 63, 64*), albeit with a delay (Figs. 3B, and 4B). We hypothesize that *Psm* ES4326, despite not causing noticeable PCD, contains other effectors that can not only produce damage-associated molecular patterns, but also be weakly recognized by immune receptors other than RESISTANT TO P. SYRINGAE 2 (RPS2), because mutating these receptors partially inhibits *Psm* ES4326/avrRpt2-mediated PCD and resistance in the presence of RPS2 (65). Different strains of pathogens carry distinct sets of effectors whose interactions with host targets may trigger other signaling events besides the one identified in this study (*63, 64, 66, 67*). Utilizing effector-triggered immunity (ETI)-inducing avirulent pathogens can simplify the study because they could more quickly induce sufficient H_2_O_2_ through ETI in local tissues to trigger SAR.

It is intriguing that a circadian clock TF (i.e., CHE) controls SAR induction in plants, raising questions about the biological significance. Since mutating the cysteine in CHE does not affect its clock function (Fig. 2, E and F, and fig. S4), CHE’s role in SAR is unlikely through the circadian clock. Previously, we showed that ETI-associated PCD is gated by the redox rhythm towards the morning (68). Therefore, CHE’s peak expression around dusk (fig. S2A) aligns with the time when the H_2_O_2_ signal, produced in the morning, reaches systemic tissues (Fig. 4B and Movies S1 and S4), sulfenylating CHE throughout the night (Fig. 3, A and B) to induce robust SAR in the morning when conditions, such as high humidity, favor many pathogens *(25, 69–71)*.

Our finding that an optimal H_2_O_2_ concentration is required to sulfenylate CHE reconciles conflicting reports on H_2_O_2_’s role in defense (*49, 50*) because higher-than-optimal H_2_O_2_ levels would inactivate CHE in inducing *ICS1* by further oxidizing CHE (Fig. 3, D and H). It is tempting to hypothesize that the sensitivity to H_2_O_2_ concentration allows plants to gauge the level of local infection to determine whether and how much to activate SAR. Therefore, our study not only identifies H_2_O_2_ as the primary mobile signal and H_2_O_2_-mediated CHE-SOH as the signal transduction mechanism in inducing systemic SA synthesis, but also conforms with previous discoveries on SAR activation after local infection (*1–3, 20*).

## Supplementary Material

Table S1-3 Tables S1 to S3

Movie S1 Movies S1 to S7

Movie S2

Movie S3

Movie S4

Movie S5

Movie S6

Movie S7

supplemental materials (methods and fig. S1-9 Materials and Methods
Figs. S1 to S9

## Data Availability

All generated materials in the manuscript are available upon request. All data are available in the main text or the supplementary materials.
