# Supplementary material for: H2O2 sulfenylates CHE linking local infection to establishment of systemic acquired resistance
Source: Science. Author manuscript; Available in PMC 2024 Nov 24. (PMC11586058; doi:10.1126/science.adj7249)
Supplement: supplemental materials (methods and fig. S1-9 — Materials and Methods Figs. S1 to S9 [file NIHMS2031248-supplement-supplemental_materials__methods_and_fig__S1-9.pdf]

Science AAAS

Supplementary Materials for

H_2O_2 sulfenylates CHE, linking local infection to the establishment of systemic acquired resistance

Lijun Cao[1,2], Sargis Karapetyan[1,2], Heejin Yoo[1,2,†], Tianyuan Chen[1,2], Musoki Mwimba[1,2], Xing Zhang[1,2], and Xinnian Dong[1,2,*]

Corresponding author: xdong@duke.edu

The PDF file includes:

Materials and Methods
Figs. S1 to S9
References (72-81)

Other Supplementary Materials for this manuscript include the following:

Tables S1 to S3
Movies S1 to S7

Materials and Methods

Plant materials

Arabidopsis thaliana, including wild type (WT), *che-2 (28)*, *che-1 (28)*, *che-3* (SALK_106694), *lhy-20 (28)*, *ald1 (24)*, *fmo1 (63)*, *sid2-2 (51)*, *rbohD (72)*, *rbohF* (*72*), *sard1-2 (27)*, and transgenic plants used in this study are all in the Columbia-0 ecotype background. Plants were grown in soil at 22 ºC under 12 hours light/12 hours dark with 60% relative humidity.

Plasmid construction and plant transformation

To generate the CHE complementation lines (*CHE-HA/che-2*), the *CHE* native promoter driving the coding sequence in pGWB513 was used to transform *che-2*. The promoter and coding sequence was PCR amplified from the genomic DNA and cloned into pDONR207 with Gateway^{TM} BP Clonase^{TM} II Enzyme Mix (ThermoFisher Scientific) and then cloned into pGWB513 with Gateway^{TM} LR Clonase^{TM} II Enzyme Mix (ThermoFisher Scientific) for plant transformation. Vectors of cysteine mutants (cysteine mutated to serine in *che*^{CS}*-HA* and cysteine mutated to tryptophan in *che*^{CW}*-HA*) were generated through PCR amplification with corresponding primers (Table S1) using pGWB513 carrying *CHE-HA* as the template, then the template vector was removed by digestion with DpnI (NEB) and the cysteine mutant vectors were recovered by re-transformation in DH5α competent cells. WT *CHE* or *che*^{CS} coding sequence was constructed into pGEX6P1-DEST-HA and used for protein expression and purification in *E. coli* (Lemo21, DE3). Native promoter-driven the coding sequence of WT *CHE* or mutant *che* cloned into pGWB513 was used for transformation of WT, *che-1* (*CCA1*$_P$*:LUC*) and *che-1 lhy-20* (*CCA1*$_P$*:LUC*), *rbohD*, *sard1-2*, *ald1*, *fmo1*, *sid2-2* mutants. For the *GLUTAREDOXIN 13* (*GRXS13*) luciferase reporter line, the 1621 bp region (including the 5' leader sequence) upstream of the start codon of *GRXS13* was PCR amplified and transferred into the pDONR207 plasmid using the BP recombination reaction. The resulting entry clone was then cloned into the destination vector pGWB535 (*73*) for obtaining the final *GRXS13p:LUC* construct using LR recombination. The final constructs were transformed into *Agrobacterium tumefaciens* strain GV3101 using the heat-shock method. All plasmids constructed were confirmed by Sanger sequencing before use. *Agrobacterium*-mediated plant transformation was performed as described (*74*).

The induction of SAR and measurement of bacterial growth

For induction of SAR, *Pseudomonas syringae* pv. *maculicola* (*Psm*) ES4326 carrying the effector gene *avrRpt2* (*Psm* ES4326/avrRpt2) (OD_{600nm} = 0.01) was inoculated into either half-leaves as the local tissues (local) and the other un-inoculated neighboring half-leaves as systemic$_{nbr}$ tissues or two lower leaves as local tissues and two upper distal leaves as systemic$_{dist}$ tissues.

For examining SAR protection against bacterial growth, 3-week-old plants were first inoculated with 10 mM $MgCl_2$ (M) or *Psm* ES4326/avrRpt2 (OD_{600nm} = 0.01). After 2 days, the systemic leaves were inoculated with *Psm* ES4326 (OD_{600nm} = 0.001). Bacterial growth was measured 3 days after the second pathogen infection. Two leaf discs (0.6 cm in diameter) were placed in one of the 8-strip tubes containing 1 metal bead and 500 μl sterilized 10 mM $MgCl_2$ and ground twice using a SPEX^{TM} SamplePrep 2010 Geno/Grinder^{TM} (ThermoFisher Scientific) at 1500

strokes/min for 30 seconds. Then, the samples were centrifuged at 2000 rpm for 1 min. 10 × dilution (6 gradient dilutions per sample) was used for each sample. 10 µl aliquots from each dilution were spread on King's B Medium. The bacteria were grown for 2 days at 30 ºC and colony-forming units were counted for each dilution. The bacteria growth was calculated based on the dilution and the area of leaf discs.

For petiole exudate (PeX)-triggered defense induction, 3-week-old plants were first inoculated with PeX collected using the method specified below. One day after, the treated or distal leaves were inoculated with *Psm* ES4326 (OD_{600nm} = 0.001) and bacterial growth was measured 3 days after the pathogen infection.

Quantitative real-time PCR

Total RNA was extracted from leaf tissues of 3-week-old plants using TRIzol, followed by DNase I (Invitrogen) treatment to remove genomic DNA contamination. The extracted RNA was then used as a template for reverse transcription using SuperScript® III Reverse Transcriptase (Invitrogen) with an oligo (dT) primer. FastStart Universal SYBR Green Master (Roche) was used in real-time PCR with primers listed in Table S1. *Ubiquitin 5* (*UBQ5*) was used as the internal control.

SA, Pip, NHP, and G3P quantification

Free SA, Pip, NHP, and G3P were measured as described (*15, 75*) with modifications. 3 to 6 leaves were collected, weighed, and ground in liquid nitrogen. The samples were then extracted in 1 ml of MeOH/H_2O (80/20, v/v), then vortexed for 1 min and incubated at 4 ºC for 10 min with rotation. The supernatant was collected after centrifugation at 4 ºC (14,000 g for 10 min). The extraction was repeated one more time before combining the two-extracted supernatants into a 2-ml tube, and then the supernatant was collected after another centrifugation. 400-800 µl of the extract was dried using an Eppendorf concentrator plus/Vacufuge® plus system at 4 °C. Then, 100 µl of a 50% methanol solution was added to the sample vials and vigorously vortexed. The solution was centrifuged at 5 °C (15,000 rpm for 5 min) and 40 µl of the resulting supernatant was transferred into the well of a 1 ml 96-well NUNC plate (ThermoFisher Scientific). After adding 10 µl internal mixture, the plate was mixed on a ThermoMixer at 1,000 rpm for 10 min, then centrifuged at 3,000 rpm for 2 min before injection into the instrument.

Samples were analyzed using the Waters TQ-S MS system (Milford, MA) with the Acquity UPLC. Software Masslynx 4.2 was used for data acquisition. The LC separation was performed on a Waters Acquity CSH phenyl-hexyl column (2.1 x 100 mm, 1.7 µm) with mobile phase A (0.1% formic acid in water) and mobile phase B (0.1% formic acid in acetonitrile). The flow rate was 0.45 ml/min. The linear gradient was as follows: 0 - 1 min, 100% A; 3 min, 50% A; 3.1 - 4.5 min, 0% A; 4.6 - 6.1 min, 100% A. The autosampler was set at 10 °C and the column was kept at 45 °C. The injection volume was 2 µl. Mass spectra were acquired under positive and negative electrospray ionization (ESI) with MRM as the detection approach. The positive mode was used for pipecolic acid (m/z 130.0 → m/z 67.0) and internal standard pipecolic acid-d_9 (m/z 139.0 → m/z 61.0). The negative mode was used for salicylic acid (m/z 137.0 → m/z 93.0) and internal standard salicylic acid-d_4 (m/z 141.0 → m/z 97.0).

The QTrap 6500+ LC-MS/MS system (Sciex, Framingham) and software Analyst 1.7.3 were used for data acquisition and analysis of N-hydroxypipecolic acid (NHP) and Glycerol-3-phosphate (G3P). The Waters I-class plus UPLC system includes a degasser, an autosampler, a column oven, and a binary pump. To measure NHP, a Waters Acquity CSH phenyl-hexyl column (2.1 x 100 mm, 1.7 µm) with mobile phase A (0.1% formic acid in water) and mobile phase B (0.1% formic acid in acetonitrile) was used. The flow rate was 0.45 ml/min. The linear gradient was as follows: 0 - 0.1 min, 100% A; 2 min, 50% A; 2.3 - 3.5 min, 0% A; 3.6 - 4.6 min, 100% A. The autosampler was set at 10 °C and the column was kept at 50 °C. The injection volume was 2 µl. Mass spectra were acquired under positive and negative electrospray ionization (ESI) with the ion spray voltage of + 2800 V and - 2000 V, respectively. The source temperature was 500 °C. The curtain gas, ion source gas 1, and ion source gas 2 were 35, 60, and 70 psi, respectively. Mass spectra were acquired under both positive and negative electrospray ionization (ESI) with Multiple reaction monitoring (MRM) as the detection approach: N-hydroxypipecolic acid (m/z 146.0 → m/z 55.0) and internal standard were measured in positive mode. To measure glycerol-3-phosphate, the separation was performed on a Thermo Accucore PFP column (2.1 x 50 mm, 2.6 µm) with mobile phase A (0.1% formic acid and 5 mM ammonia formate in water) and mobile phase B (methanol). The injection volume was 2 µl. The flow rate was 0.5 ml/min. The linear gradient was as follows: 0 - 0.1 min, 90% A; 2 min, 50% A; 2.3 - 3.5 min, 0% A; 3.6 - 4.2 min, 90% A. The autosampler was set at 10 °C and the column was kept at 50 °C. Mass spectra were acquired under negative electrospray ionization (ESI) with the ion spray voltage of - 4500 V. The source temperature was 450 °C. The curtain gas, ion source gas 1, and ion source gas 2 were 33, 55, and 60 psi, respectively. MRM was used to detect glycerol-3-phosphate (m/z 171.0 → m/z 79.0).

For the measurement of free SA, NHP, and G3P in PeX, 1 ml of petiole extract was dried and subjected to the same procedure as described above.

RASL-seq

One half-leaf from each plant was inoculated with either mock or *Psm* ES4326/avrRpt2, then three leaves from three individual plants were collected with the inoculated and uninoculated halves grouped to form one independent sample replicate for the local and systemic$_{nbr}$ tissues, respectively. We utilized 3 to 5 independent replicates for RASL-seq. Total RNA extracted using TRIzol (Ambion) was processed as described (*26*). Primers used in RASL-seq are listed in Tables S2 and S3. The raw sequencing data were first aligned to gene-specific primers. The gene counts were subjected to library size normalization and log2 transformation. The resulting gene expression matrix is provided in Tables S2 and S3. The expression matrix was then normalized with Z-Score and clustered with Average Linkage in Heatmapper (http://www.heatmapper.ca). Kendall's Tau was employed as the distance measurement method in the heatmap.

Petiole exudate collection and treatment

The petiole exudate (PeX) was collected as previously described (*76*) with modifications. Specifically, we first tried two different concentrations of *Psm* ES4326/avrRpt2 (OD_{600nm} = 0.01 and OD_{600nm} = 0.0005) to inoculate 3-week-old plant leaves, and excised the leaves at 8 and 24 hours, respectively, for PeX collection for 48 hours. After proper dilution, both inoculants

produced similar results in H_2O_2 production, CHE sulfenylation, and plant defense against *Psm* ES4326. Consequently, we used $OD_{600nm} = 0.0005$ to simulate a natural infection more closely for the experiments. Another change made in this study was the inclusion of 0.02% acetanilide in the collection solution to stabilize H_2O_2. The collected PeXs were then used to treat plants, from which leaf samples were used at 4 hours after treatment (hpt) for protein sulfenylation analysis, or at 24 hpt for quantitative real-time PCR analysis and bacterial inoculation to examine induction of resistance. 50 µl of the PeX was used to quantify H_2O_2 concentration. To remove H_2O_2 from the PeX, 0.1 mg/ml catalase (Sigma) was used. To denature catalase, the catalase solution was incubated at 95 ºC for 15 min. To rescue the catalase-treated PeX, an Amicon® Ultra-4 centrifugal filter (Millipore) was used to remove the enzyme, and 10 µM H_2O_2 was added to the collected PeX.

Chromatin immunoprecipitation (ChIP)

ChIP was performed as previously described (*77*) with minor modifications. Pierce™ Anti-HA magnetic beads (ThermoFisher Scientific) were used for immunoprecipitation (IP) as described in the manufacturer's protocol, and the primers used are listed in Table S1.

Plant treatment with SA, NHP, NAD^+, $NADP^+$, and H_2O_2

Plants were sprayed with 1 mM of SA or water and covered for two hours. Treatment with 1 mM NHP was performed as described (*15*). 0.2 mM NAD^+ and 0.4 mM $NADP^+$ were used to treat the plants as described (*10*). Different concentrations of H_2O_2 suspended in 20 mM phosphate buffer (pH 6.5) containing 0.02% acetanilide were used to inoculate the plant leaf tissues. Leaf samples were collected after the treatment at indicated times.

Luciferase imaging and circadian rhythm calculation

Independent T1 seedlings were grown on half strength of Murashige-Skoog (MS) media with 25 µg/ml hygromycin B (ThermoFisher Scientific) for 10 days for $CCA1_{P}$*:LUC* imaging or 3 to 4 weeks for $GRXS13_{P}$*:LUC* imaging at 22 ºC under 12 hours light/12 hours dark condition. Then they were sprayed with 2.5 mM luciferin (Gold Biotechnology) in 0.02% Triton X-100 (Sigma) and transferred to constant light condition for $CCA1_{P}$*:LUC* imaging or kept at normal condition for $GRXS13_{P}$*:LUC* one day before imaging. Images were taken every 1 or 2 hours with an exposure time of 20 min using a charge-coupled device camera (PIXIS 2048). The quantifications of bioluminescence intensity were performed using Image J. Periods of circadian rhythm were inferred from a sine wave as previously described (*78*).

Protein S-sulfenylation and S-sulfinylation

Protein S-sulfenylation and S-sulfinylation were measured using a biotin-switch method described previously (*48, 79, 80*) with modifications. Leaf tissues (2 g) were collected and ground in liquid nitrogen and suspended in 2.5 ml of the HEN buffer (250 mM HEPES-NaOH, pH 7.7, 1 mM EDTA, and 0.1 mM neocuproine) supplemented with 1 × protease inhibitor cocktail (Roche), 2% SDS, and 50 mM MMTS. The sample was then incubated at 50 ºC for 1 hour with shaking (Eppendorf Thermomixer R, 500 rpm) under dark conditions and the

supernatant was collected after centrifugation (4 ºC, 7830 rpm for 1 hour). The protein concentration was determined using Qubit™ Protein Assay Kit (Invitrogen) and the sample was precipitated with 20-30 volumes of acetone (pre-chilled to -20 ºC) at - 20 ºC for 2 hours. The pellet was then collected and washed with acetone (90%, -20 ºC) 3 times after centrifugation (4 ºC, 2000 rpm for 10 min).

For S-sulfenylation, the pellet was suspended with 2 - 5 ml of the HEN buffer with 1% SDS (1 ml per mg of protein) and supplied with 2 mM biotin-HPDP and 50 mM sodium arsenite and incubated at 25 ºC for 1 hour with shaking under dark condition.

For S-sulfinylation, the pellet was suspended in 2 - 5 ml HEN buffer with 0.4% SDS (1 ml per 1.5 mg of protein) supplied with 10 mM DiaAlk (AOBIOUS) and incubated at 37 ºC for 2 hours with shaking under dark condition. The solution is precipitated with 20 volumes of acetone (-20 ºC) after the reaction and centrifuged at 4 ºC (2000 rpm for 10 min). The pellet was collected and washed with acetone (90%, - 20 ºC) 3 times after centrifugation (4 ºC, 2000 rpm for 10 min). Then it was resuspended with the HEN buffer (1 ml per 1.5 mg of protein) supplied with 0.2 mM azide-biotin, 0.1 mM TBTA, and 1 mM $CuSO_4$, and incubated at 25 ºC for 2 hours with shaking under dark condition.

The subsequent steps were carried out for both S-sulfenylation and S-sulfinylation. First, 5 - 10 µl of the protein solution was set aside as input for western blot and the remaining solution was precipitated with 20 - 30 volumes of acetone at -20 ºC for 2 hours. The pellet was then collected and washed with acetone (90%, -20 ºC) 3 times after centrifugation (4 ºC, 2000 rpm for 10 min). The pellet was resuspended with 1 ml of the HEN buffer with 0.1% SDS (1 ml per 1 mg of protein) mixed with 2 ml neutralization buffer (20 mM HEPES-NaOH, pH 7.7, 1 mM EDTA, 100 mM NaCl, and 0.5% Triton X-100) and 150 µl pre-washed streptavidin-agarose. The mixture was centrifuged at 200 g for 5 seconds after incubating at 25 ºC for 2 hours. The agarose was washed with neutralization buffer with 600 mM NaCl 5 times. The agarose and the input solution were then incubated with 50 µl (per mg protein) of the Elution buffer (20 mM HEPES-NaOH, pH 7.7, 1 mM EDTA, 10 mM NaCl, and 100 mM 2-Mercaptoethanol) at 25 ºC for 1 hour or 95 ºC for 15 min. The supernatant was used for western blot analysis after mixing with 2 × SDS-PAGE buffer and 1 mM DTT. The western blot was run with Invitrogen™ NuPAGE 4 to 12%, Bis-Tris, 1.0-1.5 mm, Mini protein Gels according to the manufacturer's protocol, and the protein was blotted using anti-HA antibody (HA-Tag, 6E2, Mouse mAb #2367, Cell Signaling Technology).

For detection of sulfenylation of in vitro purified protein, 40 µg protein (80 µg/ml) was dissolved in 2.5 ml of the HEN buffer (250 mM HEPES-NaOH, pH 7.7, 1 mM EDTA, 0.1 mM neocuproine) and subjected to the same procedures described above.

Protein purification and electrophoretic mobility shift assay (EMSA)

The CHE protein fused with glutathione-S-transferase (GST) was expressed in the *E. coli* strain BL21 and purified using Pierce™ glutathione magnetic agarose (ThermoFisher Scientific). The purified protein was digested with PreScission Protease (APEXBIO) to remove the GST tag. The concentration of the protein was diluted to 80 µg/ml and treated with 25 mM DTT or H_2O_2 at 25

ºC for 30 min. The treated protein was cleaned up using a Micro Bio-Spin™ P-6 Gel Column (BIO-RAD), and then an aliquot of the sample was subjected to 5 mM sodium (meta) arsenite (m-arsenite) treatment at 25 ºC for 30 min (Water treatment was used as the control). The treated protein was cleaned up using a Micro Bio-Spin™ P-6 Gel Column, and 3 µl and 0.5 ml were used for EMSA and in vitro sulfenylation, respectively. EMSA was performed using a LightShift™ Chemiluminescent EMSA kit (ThermoFisher Scientific) following the manufacturer's protocol, and the DNA probes used were listed in Table S1.

Transient expression of *NbTCP21* in *Nicotiana benthamiana*

Transient expression of *NbTCP21-1* and *NbTCP21-2* was performed as described (*81*). Briefly, plasmids expressing HA-tagged NbTCP21-1 (pGWB14-LYX149) and NbTCP21-2 (pGWB14-LYX150) were obtained from Addgene and transformed to *Agrobacterium* strain GV3101. *Agrobacterium* cultures harboring the constructs were individually collected after overnight culture and resuspended in the inoculation buffer (10 mM $MgCl_2$, 10 mM MES and 200 µM acetosyringone), adjusted to an OD_{600nm} = 0.5, and incubated at room temperature for 2 hours. Subsequently, the suspension was used to inoculate one half-leaf of 5-week-old *N. benthamiana* to express TCP-HA. 24 hours postinoculation, the untreated half-leaf was inoculated with *Pseudomonas syringae* pv. tomato DC3000 (*Pst*) (OD_{600nm} = 0.01) to induce SAR. One day after, the half-leaf expressing TCP-HA was harvested for sulfenylation analysis.

Hydrogen peroxide (H_2O_2) quantification

Three leaf discs (0.5 cm in diameter) were collected, ground in liquid nitrogen, and suspended in 600 µl pre-cooled 20 mM phosphate buffer (pH 6.5) containing 0.02% acetanilide. After centrifugation at 4 ºC (16,200 g for 10 min), the supernatant was transferred to a new pre-cooled tube. To quantify the concentration of H_2O_2, 50 µl of the solution was used according to the protocol provided by Amplex™ Red Hydrogen Peroxide Assay kit (Invitrogen). For each measurement, an H_2O_2 standard curve (0 to 100 µM, each in a volume of 50 ul) was generated. Luminescence was captured using the Victor3 plate reader (PerkinElmer) with excitation at 531 nm and emission detection at 595 nm. The H_2O_2 concentration was determined using the standard curve and divided by the area of the leaf disc. For H_2O_2 collected from PeX, the concentration was determined directly according to the standard curve.

Live imaging of reactive oxygen species (ROS)

Live ROS images were recorded as previously described (*53*) with modifications. 3-week-old plant leaves were inoculated with 50 µM 2',7'-dichlorodihydrofluorescein diacetate (H_2DCFDA) (Millipore-Sigma) in 50 mM phosphate buffer (pH 7.4) and allowed 6-8 hours to recover. Then the inoculated plants were treated with the pathogen or mock solution, and images were taken every 20 min for 2 days using a microscope (LEICA M205 FA). Imaging data were analyzed with ImagJ (FIJI).

Statistical analysis

Two-tailed Student's t-tests and two-way ANOVA were performed using GraphPad prism 8. Sample size is described in the relevant figure legends and experiments have been performed at least twice with similar results. For SA, Pip, NHP and, G3P measurement, CHIP-qPCR, and western blots, at least 3 independent samples were used in the figures.

Fig S1

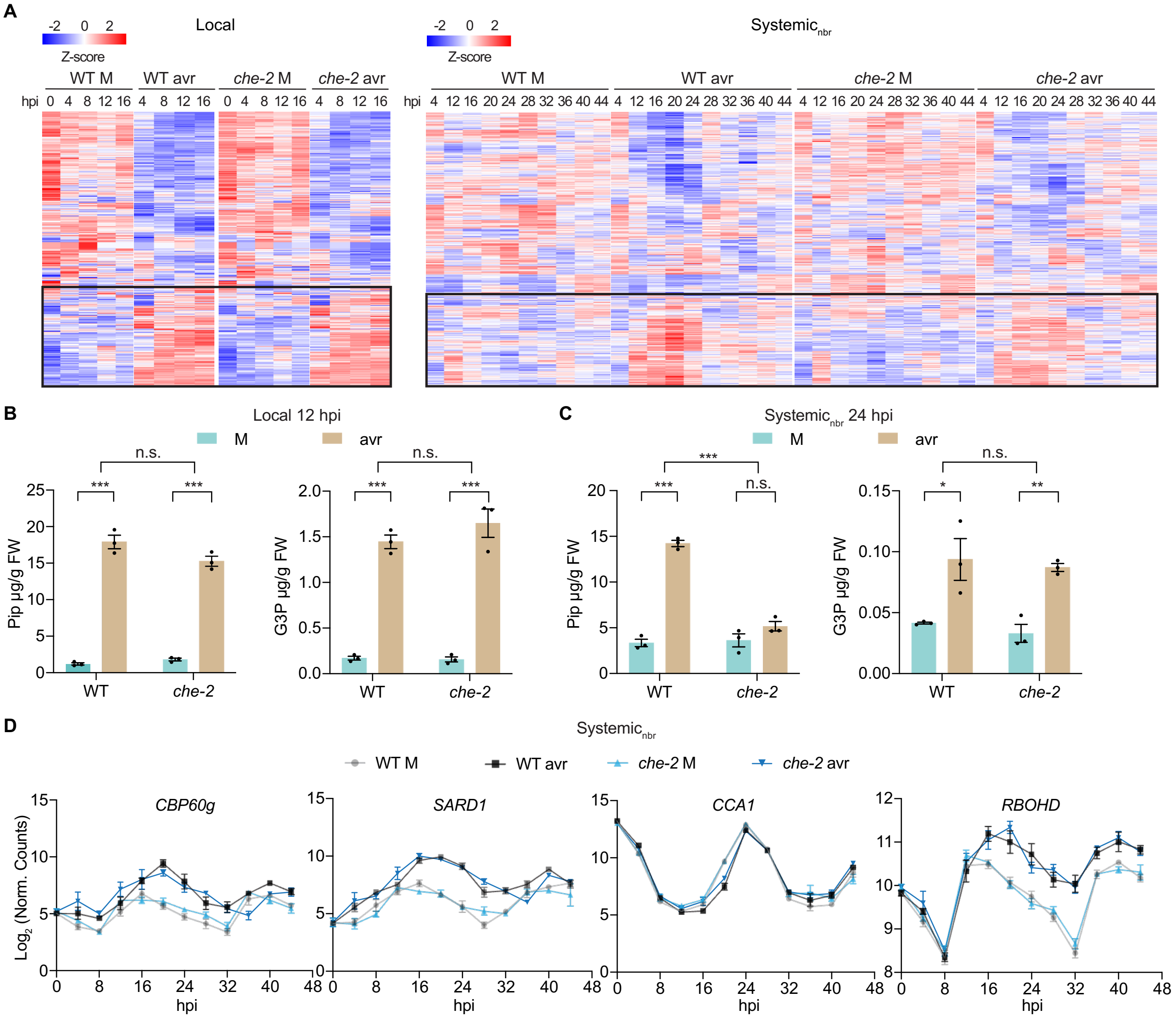

Fig. S1. CHE functions specifically in the systemic tissues during SAR. (**A**) Heatmaps of normalized RASL-seq reads in the treated (local) and untreated neighboring half leaf (systemic$_{nbr}$) tissues after mock (M; 10 mM $MgCl_2$) or *Psm* ES4326/avrRpt2 (avr; OD_{600nm} = 0.01) treatment. The gene counts were subjected to library size normalization and log2 transformation. The resulting expression matrix was then normalized with Z-Score and clustered with Average Linkage in Heatmapper (http://www.heatmapper.ca). Kendall's Tau was employed as the distance measurement method in the heatmap. Black rectangles highlight the reads of the clustered upregulated genes by the avr treatment (based on WT plants). hpi, hours postinoculation. (**B** and **C**) Levels of SA and G3P in local (B) and systemic$_{nbr}$ (C) tissues. Data are means ± SEMs (n = 3). (**D**) Normalized transcript read counts in WT and *che-2* plants collected from systemic$_{nbr}$ tissues after M or avr treatment. Data are means ± SEMs ($n \geq 3$). Significant differences were calculated using either two-tailed Student's t-tests or two-way ANOVA. ***$P < 0.001$; **$P < 0.01$; *$P < 0.05$; n.s., not significant.

Fig S2

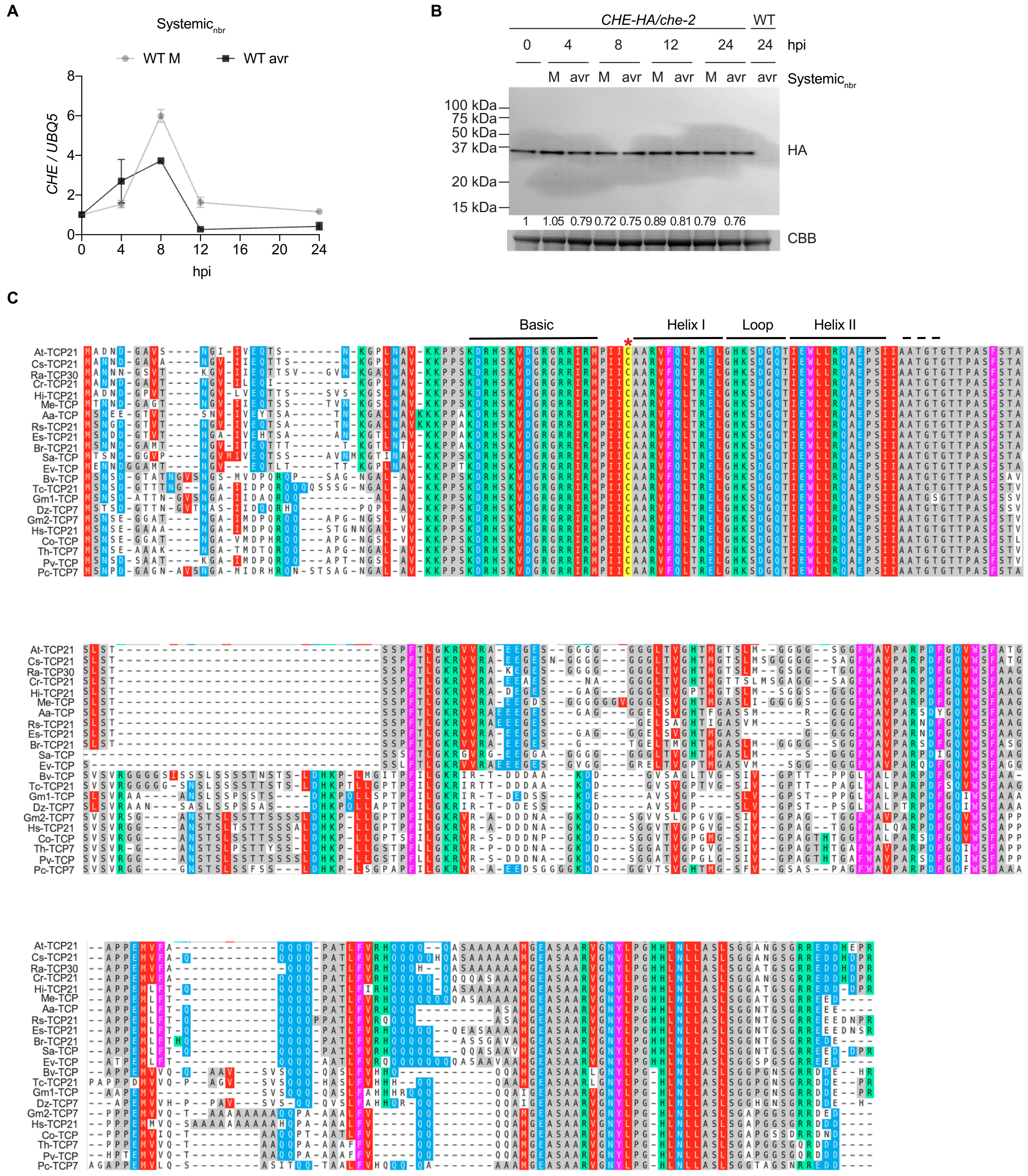

Fig. S2. CHE transcript and protein levels and conservation of the cysteine residue in CHE among plant species. (**A** and **B**) *CHE* transcript (A) and protein level (B) changes in the untreated half leaf (systemic$_{nbr}$) tissues after mock (M; 10 mM $MgCl_2$) or *Psm* ES4326/avrRpt2 (avr; OD_{600nm} = 0.01) treatment. *CHE-HA/che-2*, transgenic plants expressing WT CHE tagged with HA under its native promoter in the *che-2* mutant background. hpi, hours postinoculation.

Data are means ± SEMs. $n = 3$ for (A). (**C**) Sequence alignment of CHE homologs in plants. The red star indicates the conserved cysteine residue. *Arabidopsis thaliana* (*At*), *Camelina sativa* (*Cs*), *Rorippa aquatica* (*Ra*), *Capsella rubella* (*Cr*), *Hirschfeldia incana* (*Hi*), *Microthlaspi erraticum* (*Me*), *Arabis alpina* (*Aa*), *Raphanus sativus* (*Rs*), *Eutrema salsugineum* (*Es*), *Brassica rapa* (*Br*), *Sinapis alba* (*Sa*), *Eruca vesicaria* (*Ev*), *Bauhinia variegate* (*Bv*), *Theobroma cacao* (*Tc*), *Gossypium mustelinum* (*Gm*), *Durio zibethinus* (*Dz*), *Glycine max* (*Gm*), *Hibiscus syriacus* (*Hs*), *Corchorus olitorius* (*Co*), *Tarenaya hassleriana* (*Th*), *Phaseolus vulgaris* (*Pv*), *Prosopis cineraria* (*Pc*).

Fig S3

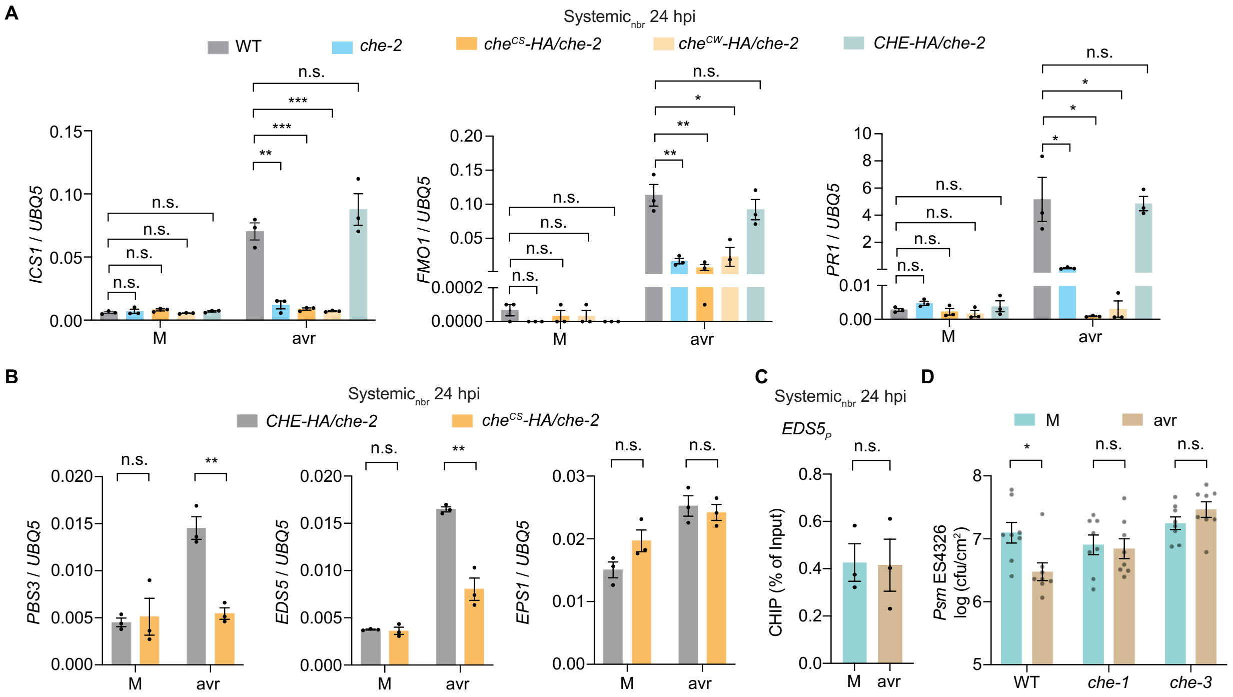

Fig. S3. Defense-related gene expression and bacterial growth in systemic tissues. (**A** and **B**) Transcriptional levels of *ICS1*, *FMO1* or *PR1* (A), and *PBS3*, *EDS5* or *EPS1* (B) in the untreated half leaf (systemic$_{nbr}$) tissues after mock (M; 10 mM $MgCl_2$) or *Psm* ES4326/avrRpt2 (avr; OD_{600nm} = 0.01) treatment. *CHE-HA/che-2*, transgenic plants expressing WT CHE tagged with HA under its native promoter in the *che-2* background. *che^{CS}-HA/che-2* and *che^{CW}-HA/che-2*, transgenic plants expressing the cysteine-to-serine and cysteine-to-tryptophan mutants. hpi, hours postinoculation. Data are means ± SEMs (n = 3). (**C**) ChIP-qPCR analysis of CHE-HA binding to the *EDS5* promoter carrying the TCP-binding site (*EDS5$_P$*) in systemic$_{nbr}$ tissues after M or avr treatment. Data are means ± SEMs (n = 3). (**D**) Bacterial growth after pathogen challenge. Plants were inoculated with M or avr 2 days before inoculation of the distal systemic tissues with *Psm* ES4326 (OD_{600nm} = 0.001), and bacterial growth was measured 3 days after the second inoculation. Data are means ± SEMs (n = 8). Significant differences were calculated using Student's t-tests. ***$P < 0.001$; **$P < 0.01$; *$P < 0.05$; n.s., not significant.

Fig S4

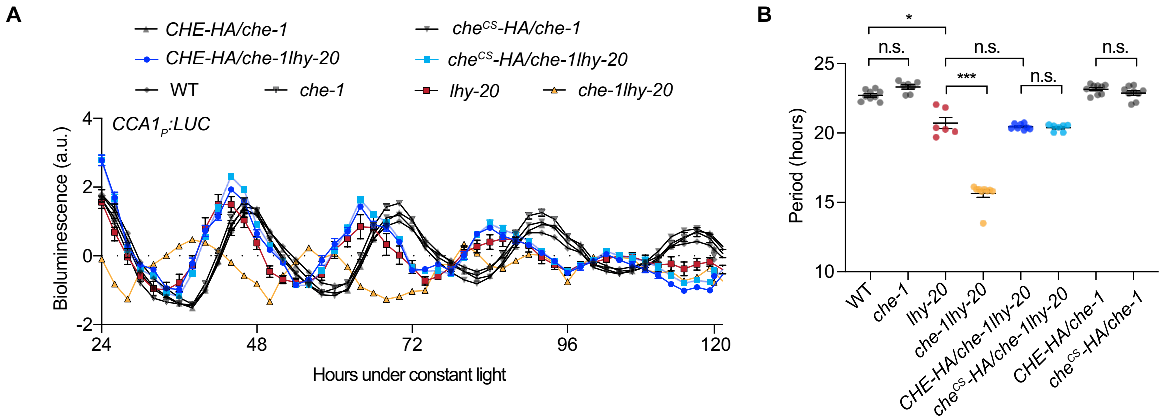

Fig. S4. The cysteine mutant can rescue the phenotype of *che-1* in the regulation of the circadian clock gene, *CCA1*. (**A**) Bioluminescence activity of *CCA1P:LUC* under constant light conditions. *CHE-HA/che-1* and *CHE-HA/che-1lhy-20*, transgenic plants expressing WT CHE tagged with HA under its native promoter in *che-1* and *che-1lhy-20* backgrounds, respectively. *che*^{CS}*-HA/che-1* and *che*^{CS}*-HA/che-1lhy-20*, transgenic plants expressing the cysteine-to-serine mutants. Data are means ± SEMs ($n \geq 6$). (**B**) Period estimates of the *CCA1P:LUC* reporter activity. Data are means ± SEMs ($n \geq 6$). Significant differences were calculated using two-tailed Student's t-tests. ***$P < 0.001$; *$P < 0.05$; n.s., not significant.

Fig S5

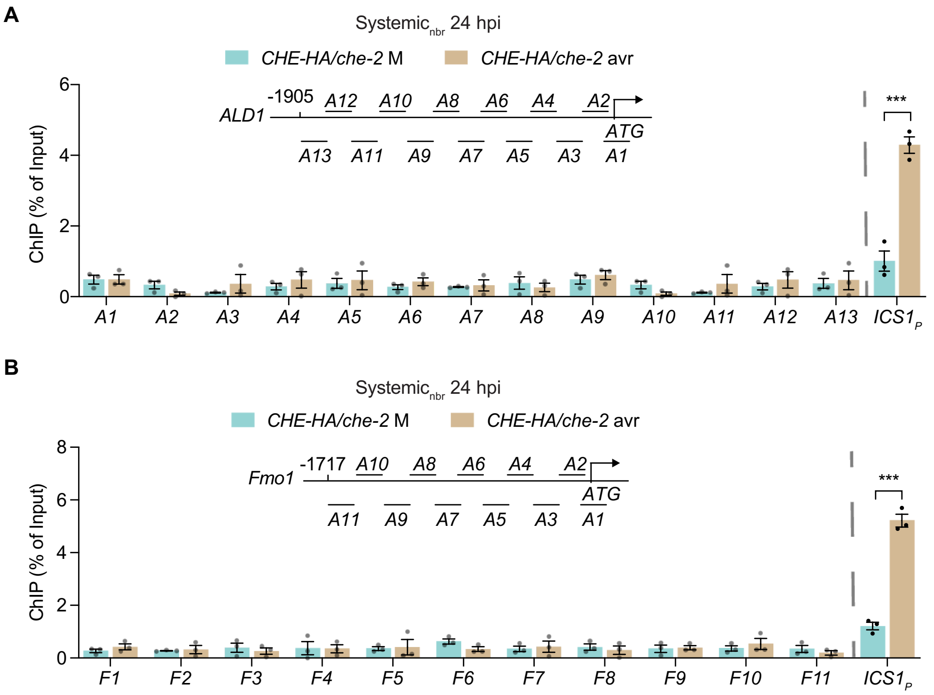

Fig. S5. CHE does not bind to the *ALD1* or *FMO1* promoter. (**A** and **B**) ChIP-qPCR analysis of CHE-HA binding to the *ALD1* promoter (A) and the *FMO1* promoter (B) in the untreated half leaf (systemic$_{nbr}$) tissues after mock (M; 10 mM $MgCl_2$) or *Psm* ES4326/avrRpt2 (avr; OD_{600nm} = 0.01) treatment. *A1* to *A13*, qPCR fragments covering the promoter sequence of *ALD1* (1905 bp upstream ATG to 46 bp downstream ATG); *F1* to *F11*, qPCR fragments covering the promoter sequence of *FMO1* (1717 bp upstream ATG to 53 bp downstream ATG); *ICS1$_P$*, the *ICS1* promoter sequence carrying the TCP-binding site. hpi, hours postinoculation. Data are means ± SEMs (n = 3). Significant differences were calculated using two-tailed Student's t-tests. ****P* < 0.001.

Fig S6

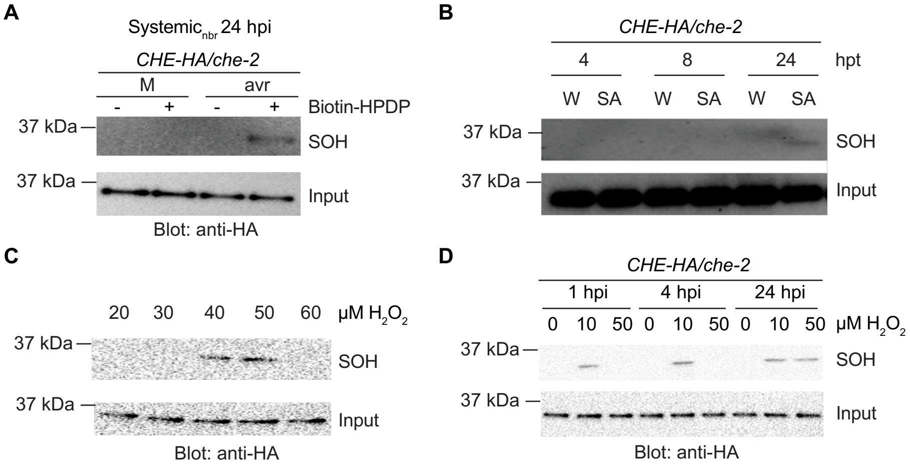

Fig. S6. Sulfenylation of CHE. (**A**) Sulfenylation (SOH) of CHE in the untreated half leaf ($systemic_{nbr}$) tissues after mock (M; 10 mM $MgCl_2$) or *Psm* ES4326/avrRpt2 (avr; OD_{600nm} = 0.01) treatment. *CHE-HA/che-2*, transgenic plants expressing WT CHE tagged with HA under its native promoter in the *che-2* mutant background. hpi, hours postinoculation. (**B**) Time-course sulfenylation (SOH) of CHE in the tissues after water (W) or 1 mM SA treatment. (**C**) In vitro sulfenylation (SOH) of CHE after treatment of the protein with different concentrations of H_2O_2. (**D**) Sulfenylation (SOH) of CHE in leaf tissues treated with different concentrations of H_2O_2.

Fig S7

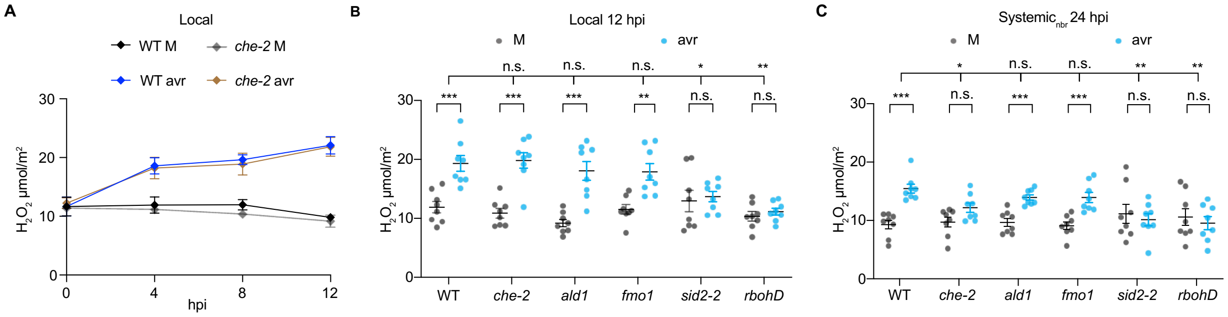

Fig. S7. H_2O_2 production after pathogen challenge. (**A**) Time-course measurement of H_2O_2 in treated leaf (local) tissues after mock (M; 10 mM $MgCl_2$) or *Psm* ES4326/avrRpt2 (avr; OD_{600nm} = 0.01) treatment. hpi, hours postinoculation. Data are means ± SEMs (*n* = 5). (**B** and **C**) Levels of H_2O_2 produced in the treated (local) (B) or the untreated half leaf (systemic$_{nbr}$) tissues (C). Data are means ± SEMs (*n* = 8). Significant differences were calculated using either two-tailed Student's t-tests or two-way ANOVA. ****P* < 0.001; ***P* < 0.01; **P* < 0.05; n.s., not significant.

Fig S8

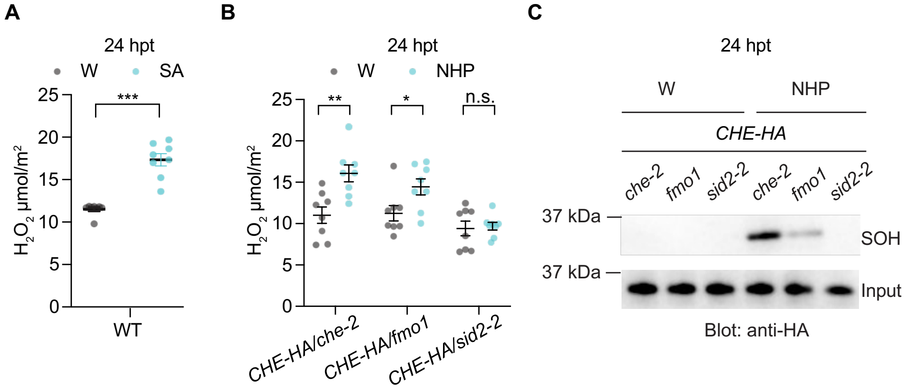

Fig. S8. Production of H_2O_2 induced by SA and NHP, and CHE sulfenylation induced by NHP. (**A** and **B**) Levels of H_2O_2 produced 24 hours after 1 mM SA (A) or NHP (B) treatment. Water (W) treatment was used as a negative control. *CHE-HA/che-2*, *CHE-HA/fmo1*, and *CHE-HA/sid2-2*, transgenic plants expressing WT CHE tagged with HA under its native promoter in *che-2, fmo1*, and *sid2-2* mutants, respectively. hpt, hours post treatment. Data are the means ± SEMs ($n = 8$). (**C**) Sulfenylation of CHE after NHP treatment in different transgenic plants. Significant differences were calculated using two-tailed Student's t-tests. $^{***}P < 0.001$; $^{**}P < 0.01$; $^{*}P < 0.05$; n.s., not significant.

Fig S9

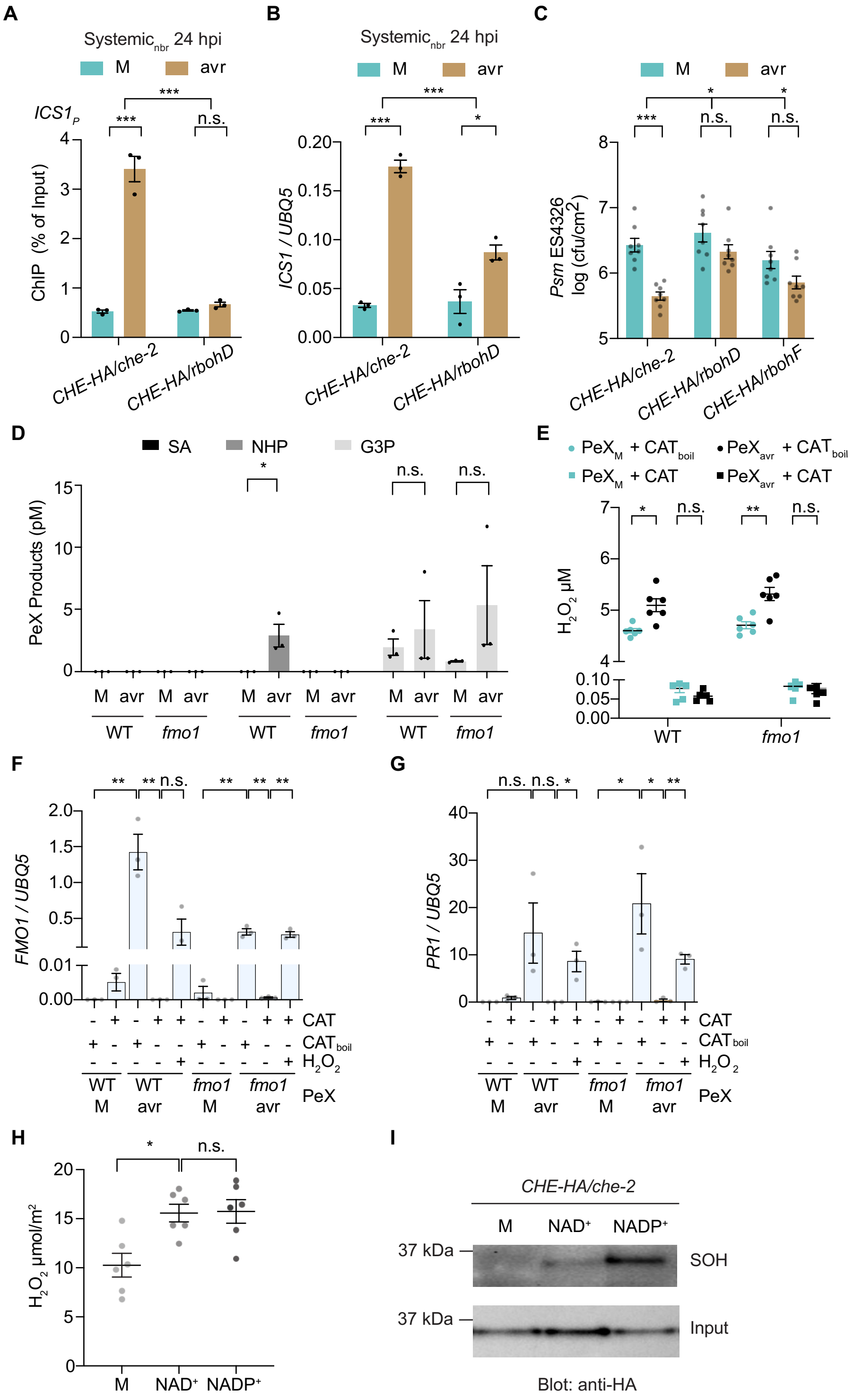

Fig. S9. H_2O_2 produced by RBOHs initiates systemic SA synthesis. (**A-C**) ChIP-qPCR of CHE-HA binding to the *ICS1* promoter TCP-binding site (*ICS1P*) (A), expression of *ICS1* (B) and bacterial growth (C) in systemic tissues after mock (M) or *Psm* ES4326/avrRpt2 (avr) treatment. *CHE-HA/che-2*, *CHE-HA/rbohD*, and *CHE-HA/rbohF*, WT CHE under its native

promoter expressed in *che-2, rbohD*, and *rbohF* mutants, respectively. Data are means ± SEMs. *n* = 3 for (A and B), *n* = 8 for (C). (**D**) SA, NHP, and G3P levels in petiole exudates (PeX) collected from WT and *fmo1* plants. Data are means ± SEMs (*n* = 3). (**E**) PeX H_2O_2 levels after treatment with catalase (CAT) or heat-denatured catalase (CAT_{boil}). Data are means ± SEMs (*n* = 6). (**F** and **G**) Expression of *FMO1* (F) and *PR1* (G) in WT plants after inoculation with PeX. Data are means ± SEMs (*n* = 3). (**H** and **I**) H_2O_2 level (H) and CHE sulfenylation (I) 4 hours after NAD^+ or $NADP^+$ treatment. Data are means ± SEMs (*n* = 6) for H. Significant differences were calculated using either two-tailed Student's t-tests or two-way ANOVA. ****P* < 0.001; ***P* < 0.01; **P* < 0.05; n.s., not significant.

References and Notes

72. M. A. Torres, J. L. Dangl, J. D. G. Jones, Arabidopsis gp91(phox) homologues AtrbohD and AtrbohF are required for accumulation of reactive oxygen intermediates in the plant defense response. *Proceedings of the National Academy of Sciences of the United States of America* **99**, 517-522 (2002). doi: 10.1073/pnas.012452499
73. T. Nakagawa *et al.*, Improved Gateway binary vectors: high-performance vectors for creation of fusion constructs in transgenic analysis of plants. *Biosci Biotechnol Biochem* **71**, 2095-2100 (2007). doi: 10.1271/bbb.70216
74. S. J. Clough, A. F. Bent, Floral dip: a simplified method for Agrobacterium-mediated transformation of Arabidopsis thaliana. *Plant Journal* **16**, 735-743 (1998). doi: 10.1046/j.1365-313x.1998.00343.x
75. H. Z. Liu, K. S. Yu, P. Kachroo, Rapid and Reliable Quantification of Glycerol-3-phosphate Using Gas Chromatography-coupled Mass Spectrometry. *Bio-Protocol* **13**, (2023). doi: 10.21769/BioProtoc.4645
76. M. Wenig *et al.*, Systemic acquired resistance networks amplify airborne defense cues. *Nature Communications* **10**, (2019). doi: 10.1038/s41467-019-11798-2
77. A. V. Gendrel, Z. Lippman, R. Martienssen, V. Colot, Profiling histone modification patterns in plants using genomic tiling microarrays. *Nature Methods* **2**, 213-218 (2005). doi: 10.1038/nmeth0305-213
78. M. Zhou *et al.*, Redox rhythm reinforces the circadian clock to gate immune response. *Nature* **523**, 472-U221 (2015). doi: 10.1038/nature14449
79. S. R. Jaffrey, S. H. Snyder, The biotin switch method for the detection of S-nitrosylated proteins. *Sci STKE* **2001**, pl1 (2001). doi: 10.1126/stke.2001.86.pl1
80. J. R. Burgoyne, P. Eaton, A rapid approach for the detection, quantification, and discovery of novel sulfenic acid or S-nitrosothiol modified proteins using a biotin-switch method. *Methods Enzymol* **473**, 281-303 (2010). doi: 10.1016/S0076-6879(10)73015-9
81. G. Xu *et al.*, One-step, zero-background ligation-independent cloning intron-containing hairpin RNA constructs for RNAi in plants. *New Phytol* **187**, 240-250 (2010). doi: 10.1111/j.1469-8137.2010.03253.x

Table S1. Primers used in this study.

Table S2. Normalized read counts and probes for RASL-seq in local tissues.

Table S3. Normalized read counts and probes for RASL-seq in $\text{systemic}_{\text{nbr}}$ tissues.

Movie S1. Live ROS imaging of the WT leaf after *Psm* ES4326/avrRpt2 inoculation (avr; OD_{600nm} = 0.01).

Movie S2. Live ROS imaging of the *fmo1* leaf after *Psm* ES4326/avrRpt2 inoculation (avr; OD_{600nm} = 0.01).

Movie S3. Live luciferase reporter imaging of the *GRXS13p:LUC* plants with half leaves inoculated with *Psm* ES4326/avrRpt2 (avr; OD_{600nm} = 0.01).

Movie S4. Live luciferase reporter imaging of the *GRXS13p:LUC* plants with whole leaves inoculated with *Psm* ES4326/avrRpt2 (avr; OD_{600nm} = 0.01).

Movie S5. Live luciferase reporter imaging of the *GRXS13p:LUC* plants with half leaves inoculated with *Psm* ES4326 (OD_{600nm} = 0.01).

Movie S6. Live luciferase reporter imaging of the *GRXS13p:LUC* plants with whole leaves inoculated with *Psm* ES4326 (OD_{600nm} = 0.01).

Movie S7. Live ROS imaging of the *rbohD* leaves after *Psm* ES4326/avrRpt2 inoculation (avr; OD_{600nm} = 0.01).
